# Comparison of Definitive Radiotherapy-Based Treatment and Surgical-Based Treatment for Locally Advanced Head and Neck Soft Tissue Sarcoma

**DOI:** 10.3390/jcm12093099

**Published:** 2023-04-24

**Authors:** Qiuji Wu, Juan Wang, Shaojie Li, Jia Liu, Yanshuang Cheng, Jieying Jin, Yahua Zhong

**Affiliations:** 1 Department of Radiation and Medical Oncology, Zhongnan Hospital of Wuhan University, Wuhan 430071, China; 2 Hubei Key Laboratory of Tumor Biological Behaviors, Zhongnan Hospital of Wuhan University, Wuhan 430071, China; 3 Hubei Cancer Clinical Study Center, Zhongnan Hospital of Wuhan University, Wuhan 430071, China

**Keywords:** radiotherapy, surgery, locally advanced, head and neck soft-tissue sarcomas, outcome

## Abstract

**Background:** Head and neck soft-tissue sarcomas are rare but aggressive malignancies. Definitive radiotherapy might be an alternative treatment choice in patients unfit for surgery with preservation of organ function and facial morphology. Whether definitive radiotherapy is comparable with surgery has not been fully demonstrated. In this study, we compared the prognosis of patients with radiotherapy-based treatment and with surgery-based treatment. **Methods:** From May 2014 to February 2021, patients with locally advanced head and neck soft-tissue sarcoma treated with either definitive radiotherapy-based treatment or radical surgery-based treatment were retrospectively enrolled. Clinical outcomes including tumor response, patients’ survival and acute treatment-related toxicities were evaluated. Kaplan–Meier curves with log-rank test were used to compare survival data. Cox regression analysis was used to explore prognostic factors. **Results:** A total of 24 patients (12 males and 12 females, 3 to 61 years old) were eligible for analysis. The median follow-up time was 49 (range: 6–96) months. In 16 patients receiving definitive radiotherapy-based treatment, 6 reached complete response. The survival curve showed that there was no statistically significant difference in overall survival (OS), distant metastasis-free survival (DMFS), loco-regional relapse-free survival (LRRFS) and progression-free survival (PFS) between the two groups of patients (*p =* 0.35, *p =* 0.24, *p =* 0.48, *p =* 0.1, respectively). COX regression analysis showed that older age was associated with poor DMFS. There was no significant difference in grade 3–4 toxicities between the two groups. **Conclusions:** In cases of contradictions to surgery, refusal to surgery or failure to complete resection, chemoradiotherapy might be an alternative treatment option.

## 1. Introduction

Head and neck soft-tissue sarcomas are rare diseases with a reported incidence of 5 cases per 100,000 per year in Europe [1]. Most sarcomas occur in the trunk and extremities, with only 5–15% in the head and neck region [2]. Head and neck sarcomas account for only 1% of head and neck malignancies [3,4]. Although much progress has been made in other malignancies, the prognosis of head and neck soft-tissue sarcomas is still poor. The 5-year overall survival rate is about 60% [1]. Treatment strategies have not evolved significantly during the past few decades.

Currently, surgery remains the standard of care for localized small tumors [5], given that negative surgical margins could be achieved [4]. However, for locally advanced diseases, combined treatments, typically involving radiotherapy, chemotherapy and possible surgery are recommended [6]. Systemic treatments with chemotherapy or best supportive care may bring some benefit for head and neck soft-tissue sarcomas patients [4], but the overall outcome is extremely dismal. Surgery is preferred for localized diseases. However, in certain cases where tumors involve critical organs such as the orbital and cranial cavity, or facial surgeries with significant deterioration to the quality of life of young patients, surgery may not be an optimal choice. In addition, surgery in these settings do not guarantee a safety margin. Definitive radiotherapy-based treatment may be a good option for these patients. Although there are no prospective data showing that definitive radiotherapy-based treatment improves the local control rate of soft tissue sarcoma, the efficacy of adjuvant radiotherapy has been reported in retrospective studies [5,7]. In addition, induction chemotherapy with consecutive radical concurrent chemoradiotherapy could be considered for a curative intent. However, to date, limited by its scarcity, there lacks a consensus of the standard treatment regimen.

We found that a part of inoperable head and neck soft tissue sarcomas were treated successfully with chemoradiotherapy. The objective of this study is to compare definitive radiotherapy-based and surgical-based treatments.

Here, we report 24 cases of locally advanced head and neck soft-tissue sarcomas that were treated with definitive radiotherapy-based treatment or radical surgery-based treatment. Our main objective was to evaluate the impact of different treatment options on the outcome and prognosis of patients with advanced head and neck soft tissue sarcomas, and to explore potential prognostic risk factors, so as to provide reference for clinical decision-making.

## 2. Methods and Materials

### 2.1. Patient Recruitment

We conducted a retrospective study with institutional head and neck soft-tissue sarcoma patient cohort. From May 2014 to June 2021, locally advanced head and neck soft-tissue sarcoma patients treated with either definitive radiotherapy-based treatment or radical surgery-based treatment were enrolled for analysis. Patients were included if they met the following criteria: (1) histologically proven non metastatic stage IIIa-IV (based on the 8th edition of the AJCC/UICC staging system [8]) head and neck soft tissue sarcoma patients; (2) received either definitive radiotherapy-based treatment or radical surgery-based treatment; (3) with adequate hematological, liver and renal functions; (4) had complete clinical and follow up data. Exclusion criteria were: (1) complications of severe medical conditions; (2) did not complete planned radiotherapy; (3) lack of follow-up data. The study protocol was approved by the Zhongnan Hospital of Wuhan University Research Ethics committee. Patient consent was obtained from each participant for data collection.

### 2.2. Treatment

#### 2.2.1. Definitive Radiotherapy-Based Treatment

Patients received intensity-modulated radiotherapy (IMRT). Briefly, 63–70 Gy in 27–33 fractions was given to the primary tumors and metastatic cervical lymph nodes (for N positive patients). Conventional fractionated radiotherapy was delivered using intensity-modulated radiotherapy (IMRT) technique. Patient immobilization, radiotherapy delivery and follow-up were conducted according to institutional protocol. For target volume delineation, surgical reports, pathological descriptions and physical examination were all reviewed; and pre-treatment MRI, CT or PET/CT images were merged and used for target volume delineation. The gross tumor volume (GTV) was defined as the visible pre-treatment extent of tumor and lymph nodes as determined by clinical examinations and image findings. The CTV included the gross tumor volume (GTV) with an addition of 1.0–1.5 cm margin. The planning target volume (PTV) included GTV or CTV with an additional 3–5 mm margin. Margins were tailored to respect natural anatomic barriers. All patients were treated using 6-MV photons. Doses to the PTV1 were 63–70 Gy/27–33 fractions and doses to the PTV2 were 54–66 Gy/27–33 fractions. Doses constrain for selected organs at risk: mean dose limits for treatment planning to the normal structures were set as follows: brain 60 Gy, ears 50 Gy, oral mucosa and oropharynx 40 Gy, tongue 40 Gy, parotid glands 26 Gy, temporomandibular joint and mandible 60 Gy, throat 40 Gy. Maximum dose limits were set as follows: crystalline lens 9 Gy, retina 50 Gy, cornea 45 Gy, optic nerve 54 Gy, optic chiasm 54 Gy, pituitary 54 Gy, spinal cord 45 Gy, ears 60 Gy, oral mucosa and oropharynx 60 Gy, tongue 60 Gy, temporomandibular joint and mandible 66 Gy, throat 60 Gy.

For patients eligible of concurrent chemotherapy, cisplatin or nedaplatin (weekly 50 mg/m^2^, or 80–100 mg/m^2^ on day 1 and day 22; according to physicians’ choice) was delivered during radiotherapy. A part of patients also received induction chemotherapy prior to radiotherapy, which was composed of 2 to 4 cycles of EI regimen (Epirubicin 60 mg/m^2^, day 1–day 2; Ifosfamide 1.8 g/m^2^, day 1–day 5; every 21 days) or AI regimen (Adriamycin 40 mg/m^2^, day 1–day 2; Ifosfamide 1.8 g/m^2^, day 1–day 5; every 21 days) or GP regimen(cisplatin or nedaplatin 75 mg/m^2^, day 1; gemcitabine 1.0 g/m^2^, day 1, day 8; every 21 days) based on the choice of practicians and the tolerance of patients.

#### 2.2.2. Radical Surgery-Based Treatment

For resected patients with negative surgical margins, postoperative chemotherapy or radiotherapy was given. Briefly, 54–70 Gy in 27–31 fractions was given to tumor bed and prophylactic cervical lymph node area. Adjuvant therapy was used for patients with poor tumor differentiation, high Ki-67 index, heavy tumor burden (T4, multiple lymph node metastasis, extracapsular lymph node invasion), which were considered risk factors of recurrence and metastasis. Adjuvant chemotherapy regimens included EI (Epirubicin 60 mg/m^2^, day 1–day 2; Ifosfamide 1.8 g/m^2^, day 1–day 5; every 21 days) regimen or VAC (Vincristine 2 mg/m^2^, Epirubicin 75 mg/m^2^, Cyclophosphamide 1.2 g/m^2^, day 1; every 21 days) regimen or CYVADIC (Cyclophosphamide 500 mg/m^2^, day 1; Vincristine 1.5 mg day 1, day 5, Adriamycin 50 mg day 1, Dacarbazine 750 mg, day 1–day 5; every 4 weeks) regimen.

### 2.3. Data Collection and Follow-Up

Demographic, clinicopathological information, treatment modalities, clinical outcomes data such as tumor response, treatment failure, patient survival and treatment-related toxicities were collected and recorded. Patients were regularly followed up, and imaging examinations were performed every 3–4 months in the first three years after treatment. Follow-up was conducted every six months from the 3rd to the 5th year and every year thereafter. The main endpoint of follow up was overall survival (OS), and the secondary endpoints were distant metastasis-free survival (DMFS), loco-regional relapse-free survival (LRRFS) and progression-free survival (PFS). OS was defined as the interval between diagnosis and death from any cause or last follow-up. DMFS was defined as the interval between diagnosis and first distant metastasis or last follow-up. LRRFS was defined as the interval between diagnosis and first loco-regional relapse or last follow-up. PFS was defined as the interval between diagnosis and first treatment failure (loco-regional recurrence or distant metastasis), death or last follow-up, whichever came first.

### 2.4. Statistical Analysis

We presented categorical variables as number of patients (percentage), and continuous variables as median (interquartile range). For the analysis of continuous variables with non-normal distributions, the Mann–Whitney U-test or the Kruskal–Wallis test was used. Analysis of categorical variables was performed using the χ^2^ test and Fisher’s exact test where applicable. The Kaplan–Meier survival method and log rank test were applied to analyze survival data. Univariate and multivariate COX regression model was employed to look for prognostic factors. Statistical analysis was conducted using R software version 3.6.1 (R Foundation for Statistical Computing, Vienna, Austria). The R packages used included ‘tableone’, ‘survival’, ‘survminer’ and ‘plyr’. For all comparisons, a *p*-value below 0.05 indicated statistical significance.

## 3. Results

### 3.1. Patients’ Characteristics

**Figure 1:**
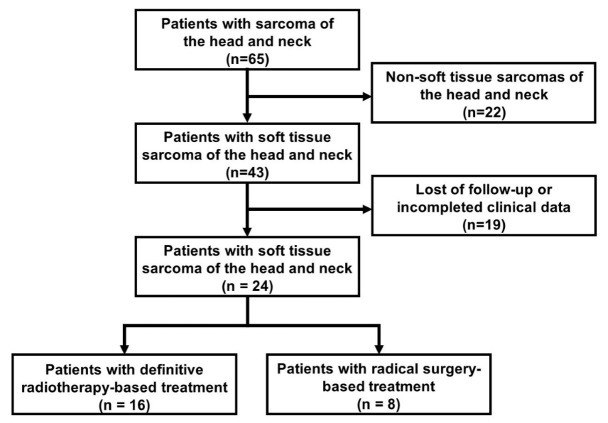
Flow diagram of patient inclusion.

In this study, 24 patients with locally advanced head and neck soft tissue sarcoma were included (Figure 1). The median age was 28.5 (range: 3–61) years old. The patients were grouped into either radical surgery-based treatment group (n = 8) or definitive radiotherapy-based treatment group (n = 16). The baseline clinicopathological characteristics of patients were shown in Table 1. Treatment protocols were shown in Table 2. There were no statistically significant differences between the two groups in terms of gender (*p =* 0.665), PS (*p =* 0.513), pathological subtype (*p =* 0.285), T stage (*p =* 0.663), N stage (*p =* 1.000), TNM stage (*p =* 1.000) and BMI (*p =* 0.098). Age (*p =* 0.018), NLR (*p =* 0.01) and PLR (*p =* 0.037) were significant differences between the two groups.

### 3.2. Abbreviations: CRT: Chemoradiotherapy; CT: Chemotherapy; RT: Radiotherapy, Objective Response Rate (ORR)

We used RECIST 1.1 criteria to evaluate the lesions of patients before and after treatment. The objective response rates of eight patients in the radical surgery-based treatment group were all complete response (CR). In the 16 patients receiving definitive radiotherapy-based treatment, 6 reached complete response (CR), 9 showed partial response (PR) and 1 showed progressive disease (PD) (Table 3).

### 3.3. Survival Outcomes

**Figure 2:**
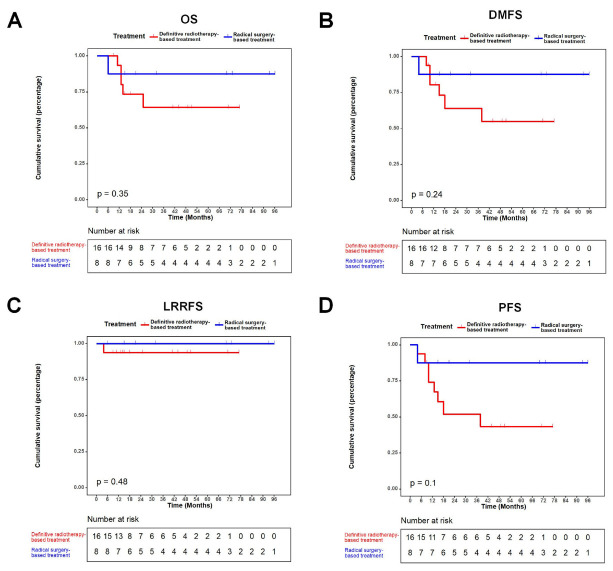
Kaplan–Meier plot. (**A**): OS. (**B**): DMFS. (**C**): LRRFS. (**D**): PFS. Abbreviations: DMFS, distant metastasis-free survival; LRRFS, locoregional recurrence-free survival; OS, overall survival; PFS, progression-free survival.

The median follow-up time of all patients was 49 (range: 6–96) months. The 3-year OS, DMFS, LRRFS and PFS in definitive radiotherapy-based treatment group and radical surgery-based treatment group were 64.2%, 63.9%, 93.8% and 51.9%, and 87.5%, 87.5%, 100% and 87.5%, respectively. In the definitive radiotherapy-based treatment group, one (6.2%) patient presented loco-regional recurrence; six (37.5%) patients presented distant metastases; five (31.3%) patients died of head and neck soft tissue sarcomas by the last follow-up. In the radical surgery-based treatment, one (12.5%) patient died of metastatic extension head and neck soft tissue sarcomas by the last follow-up; no loco-regional recurrence was observed. There was no statistically significant differences in OS (*p =* 0.350), DMFS (*p =* 0.240), LRRFS (*p =* 0.480) and PFS (*p =* 0.100) between the two groups (Figure 2 and Table 4).

### 3.4. Prognostic Analysis

We performed univariate and multivariate analysis to determine potential risk factors affecting the prognosis of patients. According to univariate Cox analysis, only age was associated with PFS (*p =* 0.043), the rest of the variables were not associated with OS, DMFS, LRRFS or PFS (all *p* > 0.05) (Table 5). During multivariate Cox analysis, no factor included was significantly associated with OS, DMFS, LRRFS, or PFS (all *p* > 0.05) (Table 6).

### 3.5. Acute Toxicities

The toxicities related to induction chemotherapy and adjuvant chemotherapy mainly included leukopenia, neutropenia, anemia, thrombocytopenia, gastrointestinal reaction and febrile neutropenia. The adverse reactions caused by concurrent radio-chemotherapy mainly included leukopenia, neutropenia, anemia, thrombocytopenia, gastrointestinal reaction, radio-mucositis and radiodermatitis. We did not observe significant difference in grade 3/4 toxicities between these two groups (All *p* > 0.05) (Table 7).

### 3.6. Representative Cases in the Definitive Radiotherapy-Based Treatment Group

**Figure 3:**
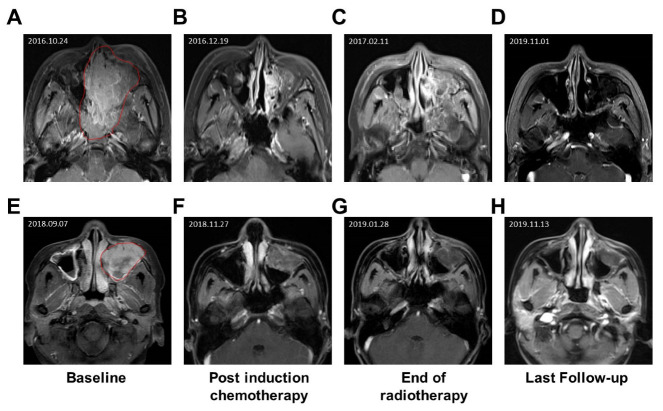
Representative cases in the definitive radiotherapy-based treatment group. (**A**–**D**) MRI images with gadolinium injection of a 22-year-old male patient (**A**) before treatment, (**B**) post induction chemotherapy, (**C**) at the end of chemoradiotherapy and (**D**) 32 months after chemoradiotherapy. (**E**–**H**) MRI images with gadolinium injection of a 24-year-old female patient (**E**) before treatment, (**F**) post induction chemotherapy, (**G**) at the end of chemoradiotherapy and (**H**) 9 months after chemoradiotherapy. The red area in (**A**,**E**) indicated tumor mass.

Figure 3 showed MRI images of tumor responses of two representative patients who received definitive radiotherapy-based treatment. Patient 1 was a 22-year-old male presented with proptosis and epistaxis for one month and was diagnosed with a sinonasal alveaolar rhabdosarcomas stage cT4N0M0, ⅢB. From 26 October 2016 to 13 December 2016, the patient underwent three cycles of chemotherapy. From 11th January 2017 to 21 February 2017, he received cisplatin-based concurrent chemoradiotherapy (PTV1: 70 Gy/30 fractions; PTV2: 60 Gy/30 fractions). He showed complete response after chemoradiotherapy (Figure 3A–D). The patient remained free of disease and was alive 71 months after treatment.

Patient 2 was a 24-year-old female presented with facial numbness, tenderness, toothache, tears; and was diagnosed with poorly differentiated sinonasal rhabdosarcomas, stage cT4N0M0, ⅢB. From 30 September 2018 to 5 December 2018, the patient underwent five cycles of chemotherapy. From 26 December 2018 to 6 February 2018, she received cisplatin-based concurrent chemoradiotherapy (PTV1: 70 Gy/31 fractions; PTV2: 63 Gy/31 fractions). Patient 2 underwent PET-CT and pathological biopsy, which confirmed that there were no residual lesions. She showed complete response after chemoradiotherapy (Figure 3E–H). The patient was free of disease 49 months after treatment.

## 4. Discussion

Head and neck soft-tissue sarcomas are very rare diseases. From 2013 to 2022, we only identified 43 patients treated in our institute, in whom 24 were eligible for analysis. Given its rarity, no consensus exists for its standard of care. For some patients with locally advanced, or positive surgical margin diseases, postoperative adjuvant radiotherapy and chemotherapy is usually adopted. Studies have shown that postoperative adjuvant radiotherapy could significantly improve local control [5] and prolong the survival time of patients [9,10]. Adjuvant systemic chemotherapies could improve the prognosis of patients, but their benefits must be weighed against related toxicities [4]. Surgery may not be the best choice for patients who are ineligible for surgery or refusing disfiguring surgery or having higher demands of quality of life. Severe medical complications, organ dysfunctions and extensive tumors involving critical organs that are hardly resectable may also restrict the use of surgery in the treatment of head and neck soft tissue sarcoma. In these cases, radiotherapy may be the first choice for clinicians and patients. We observed significant tumor regression with chemoradiotherapy in several patients who were contraindicated to surgery, intolerant to surgery, or simply refuse disfiguring surgery. Importantly, some of these patients remained free of disease years after chemoradiotherapy. Whether radiotherapy could yield similar results to surgery has not yet been determined.

Therefore, we explored whether chemoradiotherapy could be an option for those “inoperable” patients. We found that there was no significant difference in OS (*p =* 0.350), DMFS (*p =* 0.240), LRRFS (*p =* 0.480) and PFS (*p =* 0.100) between radical surgery-based treatment group and definitive radiotherapy-based treatment group. Thus, for locally advanced head and neck soft tissue sarcoma, definitive radiotherapy-based treatment might confer non-inferior outcome as compared with radical surgery-based treatment. Colville et al. showed that chemotherapy combined with radiotherapy could be used to treat sarcoma in children, and that angiosarcoma was also relatively sensitive to radiotherapy [7]. Benoit et al. reviewed 116 patients with locally/locally advanced unresectable soft tissue sarcoma and assessed response rate, local failure (LF), progression-free survival (PFS), overall survival (OS) and adverse events, revealing that radiotherapy was a safe and effective treatment yielding long term control [11]. In addition, Stephanie et al. showed in 19 children with rhabdomyosarcoma of the head and neck that radiotherapy was effective in the treatment of head and neck rhabdomyosarcoma, and the side effects were manageable [12]. Indeed, we recently conducted a retrospective study based on public data analysis using the SEER database, showing that chemoradiotherapy might confer non-inferior outcome as compared with surgery-based treatment for locally advanced rhabdomyosarcoma [13]. In the current study, we confirmed this result with a small patient size. Collectively, for patients with locally advanced head and neck soft tissue sarcoma, the efficacy of radiotherapy seems to be no worse than surgical treatment.

In addition, advanced tumors are particularly invasive and often involve critical organs of the head and face, organ-destructive and disfiguring surgery might result in declined quality of life of patients [14]. Consequently, patients may refuse surgery and choose chemoradiotherapy as an alternative treatment. In fact, several case reports showed that radiotherapy could retain organs integrity, improve the quality of life of patients, with a considerable long-term effect [15,16]. Patients with younger age, poorer tumor differentiation, larger tumor size, proximity to or involvement of vital organs and rhabdomyosarcoma were more likely to receive and benefit from radiotherapy-led therapy rather than surgery [17]. In addition, there are many cases that are unresectable or have positive incisional margins for surgery, and salvage radiotherapy is required for these patients. Richard et al. used the National Cancer Database (NCDB) to investigate overall survival (OS) in 1142 patients with positive margins after resection for head and neck sarcoma and found that salvage radiotherapy was associated with improved survival. Our study suggests that definitive radiotherapy-based treatment may be as effective as radical surgery-based treatment in patients with locally advanced head and neck sarcoma. Therefore, definitive radiotherapy-based treatment may be considered for patients with soft tissue sarcomas of the head and neck that are difficult to resect or to guarantee a negative incisal margin.

However, some studies suggest that the curative effect of radiotherapy is worse than that of surgery. A study on angiosarcoma of the head and neck showed that the survival rate of patients who underwent surgery or surgery and radiotherapy was significantly higher than that of patients who received radiotherapy alone [18]. Eeles et al. analyzed the data of 130 patients with head and neck soft tissue sarcoma and showed that the 5-year local control rate of surgery alone was higher than that of radiotherapy alone [19]. Given the limited number of patients involved in our and others’ studies and based on current evidence, we propose that surgery should still be the first choice for eligible patients, and chemoradiotherapy could only be recommended to patients who are not able to receive surgery or strongly refuse surgery.

Although radiotherapy and chemotherapy caused some adverse reactions, such as xerostomia, dental caries [20], oral mucositis, fungal infection and so on, most of them can be managed with symptomatic treatments [21]. In our study, most patients showed different degrees of myelosuppression. Six patients in the definitive radiotherapy-based treatment group showed febrile neutropenia caused by induction chemotherapy and needed hematopoietic growth factor treatment. Patients receiving radiotherapy may also present radio-mucositis and radiodermatitis. In this regard, IMRT has significantly lowed the incidence of severe radio-mucositis and radiodermatitis. Antiemetic drugs were usually given to patients with gastrointestinal reactions caused by chemotherapy. Notably, there was no significant difference in grade 3/4 adverse reactions between the two groups. The toxicities of chemoradiotherapy were well manageable regardless of the treatment protocol. No treatment-related fatal event was recorded.

Our baseline data show that there was a statistical difference in age and NLR between the two groups. This was consistent with the higher level of NLR in older patients reported in several other studies [22,23]. Neutrophils increased with age, while lymphocytes decreased with age [24]. Some studies showed that tumorigenesis was closely related to inflammatory reaction. The common markers of inflammatory reaction are platelet lymphocyte ratio (PLR), neutrophil lymphocyte ratio (NLR) and C-reaction protein (CRP). Others found that patients with soft tissue sarcoma with elevated NLR, PLR and CRP had significantly poorer survival [25,26,27,28,29,30]. In addition, high NLR was independently associated with OS deterioration in other solid tumors [31]. Neutrophils were shown to promote angiogenesis [32]. Moreover, neutrophils promoted migration and metastasis of tumor cells through degrading extracellular matrix [33]. In addition, neutrophils could inhibit cytotoxic T cell [34] and lymphokine activated killer cells [35] to promote tumor immune escape. Therefore, we included age, NLR and PLR into univariate analysis and multivariate analysis. However, our study showed that NLR and PLR were not related to OS, DMFS and PFS and age was only associated with PFS (*p =* 0.043). Such results might be related to our limited sample size.

## 5. Limitations

There were several limitations in our research. First, the sample size was small. Because patients with head and neck soft tissue sarcoma are rare, we collected follow-up data of only 43 patients from 2013 to 2022. Finally, only 24 patients met the inclusion criteria. Some patients were excluded because of insufficient information or because they did not meet our inclusion criteria. Second, our study was a single-center retrospective study, in which it would not be possible to avoid biases, such as selection bias and recall bias. A larger scale prospective study is expected to further illustrate the effect of definitive radiotherapy-based treatment. Finally, the lack of systematic analysis of patients’ quality of life and treatment satisfaction in our study was also a shortcoming.

## 6. Conclusions

In cases of contradictions to surgery, refusal of surgery or failure to complete resection, chemoradiotherapy might be an alternative treatment option. However, given the limited number of patients, the results might still be immature but only indicative and the conclusion should not be over-interpreted. The choice of treatment should be based on the experience of doctors and the wishes of patients. Further study is needed to identify patients that would mostly benefit from definitive radiotherapy.

## Figures and Tables

**Table 1:** Baseline characteristics.

Characteristics	Definitive Radiotherapy-Based Treatment (n = 16)	Radical Surgery-Based Treatment (n = 8)	*p*-Value
Gender			0.665
Male (%)	9 (56.2)	3 (37.5)	
Female (%)	7 (43.8)	5 (62.5)	
Age (years)	31.00 [24.75, 45.00]	21.00 [15.25, 26.75]	0.018
PS score (%)			0.513
0	3 (18.8)	0 (0.0)	
1	13 (81.2)	8 (100.0)	
Pathological subtype (%)			0.285
Rhabdomyosarcoma	8 (50.0)	2 (25.0)	
Fibrosarcoma	1 (6.2)	3 (37.5)	
Synovial sarcoma	3 (18.8)	2 (25.0)	
Undifferentiated sarcoma	2 (12.5)	0 (0.0)	
Others	2 (12.5)	1 (12.5)	
T * (%)			0.663
T2	6 (37.5)	2 (25.0)	
T3	5 (31.2)	2 (25.0)	
T4	5 (31.2)	4 (50.0)	
N * (%)			1
N0	6 (37.5)	3 (37.5)	
N1	10 (62.5)	5 (62.5)	
TNM stage * (%)			1
III	6 (37.5)	3 (37.5)	
IV	10 (62.5)	5 (62.5)	
BMI (kg/m^2^)	22.90 [20.82, 23.52]	19.23 [18.27, 21.03]	0.098
WBC (10^9^/L)	5.96 [4.82, 6.54]	6.06 [4.65, 6.74]	1
RBC (10^12^/L)	4.06 [3.67, 4.53]	3.80 [3.52, 4.55]	0.501
Hb (mmol/L)	123.15 [106.33, 132.15]	113.40 [107.25, 127.52]	0.624
PLT (10^9^/L)	230.00 [219.00, 303.00]	237.50 [197.25, 321.50]	0.83
NLR	2.46 [1.96, 3.55]	1.42 [1.13, 1.76]	0.01
PLR	173.07 [151.29, 227.62]	124.56 [109.31, 146.83]	0.037

Note: Data are presented as number of patients (%) or median [IQR]. Abbreviations: BMI: body mass index; CT: chemotherapy; Hb: hemoglobin; NLR: neutrophil lymphocyte ratio; PLR: platelet lymphocyte ratio; PLT: platelet; PS: performance status; RBC: red blood cell; WBC: white blood cell. * According to AJCC cancer staging system, 8th edition.

**Table 2:** Treatment protocols.

	Definitive Radiotherapy-Based Treatment (n = 16)		Radical Surgery-Based Treatment (n = 8)	
Treatment protocols (%)				
	RT alone	3 (18.8)	Surgery alone	0 (0)
	Induction CT + RT	3 (18.8)	Surgery + adjuvant CT	1 (12.5)
	Induction CT + concurrent CRT	8 (50.0)	Surgery + adjuvant CRT	3 (37.5)
	Concurrent CRT	1 (6.3)	Surgery + adjuvant RT	4 (50.0)

Note: Data are presented as number of patients (%).

**Table 3:** Objective response rates (ORR) by assess patients’ lesions.

	Definitive Radiotherapy-Based Treatment (n = 16)	Radical Surgery-Based Treatment (n = 8)
ORR (%)		
CR	6 (37.5)	8 (100.0)
PR	9 (56.3)	0 (0)
SD	0 (0)	0 (0)
PD	1 (6.3)	0 (0)

Note: ORR was assessed by nasopharyngeal magnetic resonance imaging at review two months after completion of treatment. Data are presented as number of patients (%). Abbreviations: ORR: Objective response rate; CR: complete response; PR: partial response; PD: progressive disease.

**Table 4:** Patient survivals.

	Definitive Radiotherapy-Based Treatment (n = 16)	Radical Surgery-Based Treatment (n = 8)	*p*-Value
Overall survival			0.350
3-year OS rate (95%CI)	0.642 (0.429–0.959)	0.875 (0.673–1.000)	
Distant metastasis-free survival			0.240
3-year DMFS rate (95%CI)	0.639 (0.426–0.960)	0.875 (0.673–1.000)	
Loco-regional recurrence-free survival			0.480
3-year LRRFS rate (95%CI)	0.938 (0.826–1.000)	1 (1–1)	
Progression-free survival			0.100
3-year PFS rate (95%CI)	0.519 (0.313–0.862)	0.875 (0.673–1.000)	

Abbreviations: CI: confidence interval; DMFS, distant metastasis-free survival; LRRFS, locoregional recurrence-free survival; OS, overall survival; PFS, progression-free survival.

**Table 5:** Univariate analyses of the survival prognostic factors in the advanced head and neck soft tissue sarcoma patients.

Characteristics	OS HR (95% CI)	*p*-Value	DMFS HR (95% CI)	*p*-Value	PFS HR (95% CI)	*p*-Value
Gender (Female)	0.449 (0.082–2.462)	0.356	0.359 (0.069–1.858)	0.222	0.396 (0.098–1.589)	0.191
Age(years)	1.014 (0.96–1.07)	0.62	1.018 (0.97–1.068)	0.471	1.051 (1.002–1.102)	0.043
PS (1)	0.671 (0.078–5.784)	0.717	0.797 (0.095–6.673)	0.834	0.516 (0.106–2.502)	0.411
BMI (kg/m^2^)	1.204 (0.928–1.563)	0.163	1.195 (0.938–1.522)	0.15	1.148 (0.934–1.411)	0.191
NLR	1.18 (0.9–1.549)	0.231	1.143 (0.875–1.494)	0.328	1.207 (0.974–1.497)	0.086
PLR	0.998 (0.989–1.007)	0.613	1 (0.993–1.006)	0.885	1.001 (0.997–1.005)	0.592
T (T3-T4)	0.866 (0.157–4.765)	0.869	1.237 (0.237–6.451)	0.8	0.516 (0.137–1.937)	0.327
N (N1)	3.642 (0.424–31.269)	0.239	1.815 (0.35–9.409)	0.478	2.813 (0.581–13.619)	0.199
Treatment (Surgery)	0.374 (0.044–3.207)	0.37	0.298 (0.036–2.483)	0.263	0.208 (0.026–1.669)	0.139
Induction CT (Yes)	1.41 (0.282–7.05)	0.676	1.108 (0.245–5.008)	0.894	1.048 (0.279–3.931)	0.945
Concurrent CT (Yes)	0.766 (0.14–4.208)	0.759	0.682 (0.132–3.527)	0.648	0.763 (0.19–3.061)	0.703
Adjuvant CT (Yes)	1.247 (0.144–10.77)	0.841	1.011 (0.121–8.443)	0.992	0.701 (0.087–5.623)	0.738

Abbreviations: CT: chemotherapy; DMFS, distant metastasis-free survival; LRRFS, locoregional recurrence-free survival; NLR: neutrophil lymphocyte ratio; OS, overall survival; PFS, progression-free survival; PS: performance status; PLR: platelet lymphocyte ratio.

**Table 6:** Multivariate analyses of the prognostic predictors of survival in the advanced head and neck soft tissue sarcoma patients.

Characteristics	OS HR (95% CI)	*p*-Value	DMFS HR (95% CI)	*p*-Value	PFS HR (95% CI)	*p*-Value
Age (years)	1.015 (0.925–1.113)	0.759	1.029 (0.949–1.115)	0.491	1.074 (0.997–1.157)	0.059
PS (1)	0.558 (0.033–9.48)	0.686	0.639 (0.059–6.94)	0.713	0.785 (0.119–5.189)	0.802
NLR	1.484 (0.958–2.299)	0.077	1.191 (0.829–1.712)	0.344	1.236 (0.863–1.77)	0.247
PLR	0.99 (0.974–1.006)	0.204	0.996 (0.987–1.005)	0.368	1 (0.994–1.007)	0.924
T (T3-T4)	1.367 (0.123–15.209)	0.799	3.063 (0.333–28.16)	0.323	1.435 (0.243–8.464)	0.69
N (N1)	3.053 (0.279–33.428)	0.361	1.87 (0.311–11.243)	0.494	4.961 (0.762–32.316)	0.094
Treatment (Surgery)	0.455 (0.035–5.888)	0.547	0.344 (0.031–3.808)	0.384	0.52 (0.049–5.568)	0.589

Abbreviations: DMFS, distant metastasis-free survival; LRRFS, locoregional recurrence-free survival; NLR: neutrophil lymphocyte ratio; OS, overall survival; PFS, progression-free survival; PS: performance status; PLR: platelet lymphocyte ratio.

**Table 7:** Grade 3/4 acute toxicity.

Variables	Definitive Radiotherapy-Based Treatment (n = 16)	Radical Surgery-Based Treatment (n = 8)	*p*-Value
Leukocytopenia	2 (12.5)	1 (12.5)	1
Neutropenia	2 (12.5)	1 (12.5)	1
Anemia	1 (6.2)	1 (12.5)	1
Thrombocytopenia	3 (18.8)	0 (0.0)	0.513
ALT increase	0 (0.0)	0 (0.0)	1
AST increase	0 (0.0)	0 (0.0)	1
Total bilirubin increase	0 (0.0)	0 (0.0)	1
Gastrointestinal reaction	3 (18.8)	0 (0.0)	0.513
Radio-mucositis	2 (12.5)	1 (12.5)	1
Radiodermatitis	2 (12.5)	2 (25.0)	0.846

Note: Data are presented as number of patients (%).

## Data Availability

The data presented in this study are available on request from the corresponding author.

## Abbreviations

CR: complete response, CRP: C-reaction protein, DMFS: distant metastasis-free survival, LRRFS: loco-regional relapse-free survival, NCDB: National Cancer Database, NLR: neutrophil lymphocyte ratio, ORR: objective response rate, OS: overall survival, PD: progressive disease, PLR: platelet lymphocyte ratio, PR: partial response, PS score: performance status score, PFS: progression-free survival.
